# Clinical and imaging features of interstitial lung disease in cancer patients treated with trastuzumab deruxtecan

**DOI:** 10.1007/s10147-023-02414-x

**Published:** 2023-10-03

**Authors:** Tomohisa Baba, Masahiko Kusumoto, Terufumi Kato, Yasuyuki Kurihara, Shinichi Sasaki, Katsunori Oikado, Yoshinobu Saito, Masahiro Endo, Yutaka Fujiwara, Hirotsugu Kenmotsu, Masafumi Sata, Toshimi Takano, Ken Kato, Koji Hirata, Tomomi Katagiri, Hanako Saito, Kazuyoshi Kuwano

**Affiliations:** 1 Department of Respiratory Medicine, Kanagawa Cardiovascular and Respiratory Center, 6-16-1 Tomiokahigashi, Kanazawa-ku, Yokohama-shi, Kanagawa 236-0051 Japan; 2 Department of Diagnostic Radiology, National Cancer Center Hospital, 5-1-1 Tsukiji, Chuo-ku, Tokyo, 104-0045 Japan; 3 Department of Thoracic Oncology, Kanagawa Cancer Center, 2-3-2 Nakao, Asahi-ku, Yokohama-shi, Kanagawa 241-8515 Japan; 4 Department of Radiology, St. Luke’s International Hospital, 9-1 Akashi-cho, Chuo-ku, Tokyo, 104-8560 Japan; 5 Department of Respiratory Medicine, Juntendo University Urayasu Hospital, 2-1-1 Tomioka, Urayasu-shi, Chiba 279-0021 Japan; 6 Department of Diagnostic Imaging, The Cancer Institute Hospital of Japanese Foundation for Cancer Research, 3-8-31 Ariake, Koto, Tokyo, 135-8550 Japan; 7 Department of Pulmonary Medicine and Oncology, Nippon Medical School, 1-1-5 Sendagi, Bunkyo-ku, Tokyo, 113-8602 Japan; 8 Division of Diagnostic Radiology, Shizuoka Cancer Center, 1007 Shimonagakubo, Nagaizumi-cho, Sunto-gun, Shizuoka 411-8777 Japan; 9 Department of Thoracic Oncology, Aichi Cancer Center Hospital, 1-1 Shikoden, Chikusa-ku, Nagoya-shi, Aichi 464-8681 Japan; 10 Division of Thoracic Oncology, Shizuoka Cancer Center, 1007 Shimonagakubo, Nagaizumi-cho, Sunto-gun, Shizuoka, 411-8777 Japan; 11 Division of Respiratory Medicine, Department of Internal Medicine, Jichi Medical University, 3311-1 Yakushiji, Shimotsuke-shi, Tochigi 329-0498 Japan; 12 Breast Medical Oncology Department, The Cancer Institute Hospital of Japanese Foundation for Cancer Research, 3-8-31 Ariake, Koto, Tokyo, 135-8550 Japan; 13 Department of Head and Neck, Esophageal Medical Oncology, National Cancer Center Hospital, 5-1-1 Tsukiji, Chuo-ku, Tokyo, 104-0045 Japan; 14 Clinical Safety and Pharmacovigilance Division, Medical Safety Department, Daiichi Sankyo Co., Ltd., 3-5-1, Nihonbashi Honcho, Chuo-ku, Tokyo, 103-8426 Japan; 15 Division of Respiratory Diseases, Department of Internal Medicine, The Jikei University School of Medicine, 3-25-8 Nishi-Shinbashi, Minato-ku, Tokyo, 105-8461 Japan

**Keywords:** Adverse event, Interstitial lung disease, Drug-related pneumonitis, Trastuzumab deruxtecan, Post-marketing, Computed tomography

## Abstract

**Background:**

Interstitial lung disease/pneumonitis (ILD/pneumonitis) has been identified as a drug-related adverse event of special interest of trastuzumab deruxtecan (T-DXd), but there were a few reports of T-DXd-related ILD/pneumonitis in clinical practice.

**Methods:**

Between May 25, 2020 (the launch of T-DXd in Japan) and February 24, 2022, there were 287 physician-reported potential ILD/pneumonitis cases from the Japanese post-marketing all-case surveillance. By February 27, 2022, an independent adjudication committee assessed 138 cases and adjudicated 130 cases as T-DXd-related ILD/pneumonitis. The clinical features and imaging characteristics of these cases were evaluated.

**Results:**

The majority of adjudicated T-DXd-related ILD/pneumonitis cases were grade 1 or 2 (100/130, 76.9%). The most common radiological pattern types observed were organizing pneumonia patterns (63.1%), hypersensitivity pneumonitis patterns (16.9%), and diffuse alveolar damage (DAD) patterns (14.6%). Eleven cases (8.5%) from 130 resulted in death; the majority of these (8/11, 72.7%) had DAD patterns. The overall proportion of recovery (including the outcomes of recovered, recovered with sequelae, and recovering) was 76.9%, and the median time to recovery was 83.5 days (interquartile range: 42.25–143.75 days). Most cases (59/71, 83.1%) that were treated with corticosteroids were considered responsive to treatment.

**Conclusions:**

This is the first report to evaluate T-DXd-related ILD/pneumonitis cases in clinical practice. Our findings are consistent with previous reports and suggest that patients with DAD patterns have poor outcomes. Evaluation of a larger real-world dataset may further identify predictors of clinical outcome.

**Supplementary Information:**

The online version contains supplementary material available at 10.1007/s10147-023-02414-x.

## Introduction

Trastuzumab deruxtecan (T-DXd), also known as DS-8201a, is an antibody–drug conjugate that comprises an anti-human epidermal growth factor receptor 2 (HER2) monoclonal antibody linked by a tetrapeptide-based cleavable linker to a novel cytotoxic topoisomerase I inhibitor payload [1, 2]. T-DXd has shown potent anti-tumor efficacy for the treatment of HER2-expressing or mutant cancers [3–9]. A potentially life-threatening adverse event (AE) associated with T-DXd is interstitial lung disease/pneumonitis (ILD/pneumonitis), and a pooled analysis of nine T-DXd studies reported that ILD/pneumonitis occurred in 15.4% of patients treated with T-DXd [10]. Although most ILD/pneumonitis cases were mild (grade 1 or 2), ILD/pneumonitis resulted in death in 2.2% of patients. Additionally, the risk of drug-related ILD/pneumonitis for Japanese patients treated with T-DXd was significantly higher than for patients from other countries (hazard ratio: 2.08, 95% confidence interval: 1.45–2.98) [10]. However, the number of patients enrolled in clinical trials is limited and may not reflect the diverse range of patient profiles found in clinical practice. Therefore, there was consensus that a larger real-world study could provide additional information.

As part of the approval conditions of T-DXd, surveillance of all T-DXd-treated patients in Japan was conducted. An independent ILD adjudication committee (ILD-AC) was established and assessed all physician-reported ILD/pneumonitis cases. In this report, we evaluated the clinical features and imaging characteristics of ILD/pneumonitis reported from this Japanese nationwide post-marketing surveillance program of T-DXd.

## Patients and methods

### Study design

Physician-reported potential ILD/pneumonitis cases during or after T-DXd treatment for HER2-positive metastatic breast or gastric cancer were identified from the Japanese nationwide post-marketing all-case surveillance conducted from May 25, 2020, when T-DXd was launched, until February 24, 2022.

The surveillance was conducted in accordance with the Good Post-Marketing Study Practice regulations. Written informed consent from patients and institutional review board approval were not required for the surveillance. The breast cancer survey and the gastric cancer survey were registered at the Japan Registry of Clinical Trials (https://jrct.niph.go.jp/) under the identifiers jRCT1080225197 and jRCT2001200001, respectively.

### Patients

All patients who were treated with T-DXd in clinical practice after the launch of this drug were enrolled in this surveillance. All potential ILD/pneumonitis cases occurring during or after T-DXd treatment were reported by physicians.

### Assessment of ILD/pneumonitis

The ILD-AC was established in May 2020 to independently, consistently, and retrospectively assess all physician-reported ILD/pneumonitis cases from the post-marketing clinical use of T-DXd. The committee consisted of four radiologists, eight pulmonologists, a breast cancer expert, and a gastric cancer expert. A radiologist and a pulmonologist each independently evaluated the radiographs for each potential ILD/pneumonitis case when the documentation submitted by the physicians was considered sufficient for adjudication. A pulmonologist independently evaluated the clinical data, including symptoms, clinical course, and any available relevant test results. After independent evaluation by the radiologists and pulmonologists, the committee assessed these evaluations and reviewed chest computed tomography (CT)/X-ray images and the clinical data to determine whether the event was T-DXd-related ILD/pneumonitis based on member consensus.

If the case was adjudicated as T-DXd-related ILD/pneumonitis, the following items were evaluated: date of ILD/pneumonitis onset; common terminology criteria for AE (CTCAE) grades at the time of ILD/pneumonitis onset and the most severe grade reached; the outcome of ILD/pneumonitis (Table 1); responsiveness to corticosteroids (if the patient had received them); and whether ILD/pneumonitis led to patient death if the patient died. To ensure that the outcomes of ILD/pneumonitis events reported in a post-marketing setting were evaluated consistently, the definition of ILD/pneumonitis event outcomes was discussed and agreed by the ILD-AC and the study sponsor (Table 1). Response to corticosteroids was determined by the ILD-AC pulmonologists by assessment of the clinical course and the radiological images.

**Table 1:** Definitions of ILD/pneumonitis outcomes

Category	Definition
Recovered or resolved	The patient meets both of the following criteria: 1. Symptoms have fully resolved 2. New abnormal shadows on chest CT have fully disappeared or only slight shadows are present that are considered not to affect respiratory function. If only chest X-ray images are available, then abnormal shadows have fully disappeared, or only slight shadows are present that are considered not to affect respiratory function
Recovering or resolving	The patient meets both of the following criteria: 1. Symptoms have fully resolved or have improved 2. New abnormal shadows on chest CT have improved; shadows (not considered scar-like lesions) are still present. If only chest X-ray images are available, then abnormal shadows have improved
Recovered or resolved with sequelae	The patient meets either of the following criteria: 1. Symptoms have improved but no further improvement is expected; the patient has respiratory difficulties that impact their daily living (e.g., requiring chronic oxygen therapy) 2. New abnormal shadows on chest CT have improved but no further improvement is expected; scar-like lesions which are considered to impact respiratory function are present. If only chest X-ray images are available, then abnormal shadows have improved but no further improvement is expected, and abnormal shadows that are considered to affect respiratory function are present
Not recovered or not resolved	The patient has no symptom improvement or improvement on chest CT or chest X-ray images
Fatal	Death caused by ILD/pneumonitis

*CT* computed tomography, *ILD/pneumonitis* interstitial lung disease or pneumonitis

Assessment of the ILD/pneumonitis imaging pattern for each case was performed by two or more radiologists retrospectively evaluating chest CT images from adjudicated ILD/pneumonitis cases using a similar process as used in T-DXd clinical trials. One radiologist pre-read CT images and selected a pattern; then two or more radiologists read CT images in a consensus read and agree on a pattern. Images were classified as per the following categories: diffuse alveolar damage (DAD) pattern, organizing pneumonia (OP) pattern, hypersensitivity pneumonitis (HP) pattern, non-specific interstitial pneumonia (NSIP) pattern, and others [11]. The CT images were evaluated at the onset of ILD/pneumonitis and after onset to evaluate any changes in pattern type over time. Data were summarized by number of patients, percentage, median, and range or interquartile range (IQR).

## Results

### Patient disposition and characteristics

**Fig. 1:**
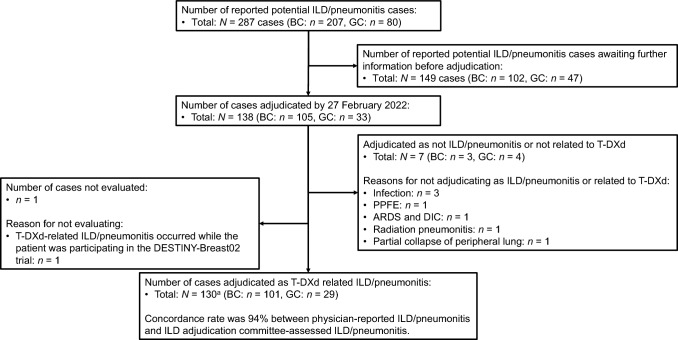
Flow diagram of ILD/pneumonitis cases. Approximately 3000 patients (1830 patients with breast cancer and 1170 patients with gastric cancer) were estimated to have initiated T-DXd treatment in Japan between May 25, 2020 and February 24, 2022. ^a^Reported terms coded by preferred terms (PTs) using MedDRA/J version 25.0: pneumonitis, interstitial lung disease, organizing pneumonia, radiation pneumonitis, and lung disorder. *ARDS* acute respiratory distress syndrome, *BC* breast cancer, *DIC* disseminated intravascular coagulation, *ILD/pneumonitis* interstitial lung disease or pneumonitis, *GC* gastric cancer, *PPFE* pleuroparenchymal fibroelastosis, *T-DXd* trastuzumab deruxtecan

The patient flow is shown in Fig. 1. Between May 25, 2020 and February 24, 2022, approximately 3000 patients (breast cancer: *n* = 1830, gastric cancer: *n* = 1170) began treatment with T-DXd; 287 of these cases were reported by their physician as potential T-DXd-related ILD/pneumonitis. A total of 138 cases were assessed by the ILD-AC as of February 27, 2022. Among these, 7/138 cases were adjudicated as either not ILD/pneumonitis or ILD/pneumonitis that was not related to T-DXd treatment. One T-DXd-related ILD/pneumonitis case was excluded from this report as the ILD/pneumonitis occurred while the patient was participating in a T-DXd clinical trial prior to marketing approval. A total of 130 cases adjudicated as T-DXd-related ILD/pneumonitis (101 patients with breast cancer and 29 patients with gastric cancer) were evaluated in this report. For 24.6% of cases (32/130), the ILD-AC considered the time of onset to be earlier than the physicians’ notation (median difference: 29.5 days [range: 1–276 days]).

The characteristics of patients with T-DXd-related ILD/pneumonitis and their CT findings prior to initiating T-DXd treatment, as reviewed by the ILD-AC radiologists, are summarized in Table 2. The median age of patients was 64.5 years (patients with breast cancer: 62 years, patients with gastric cancer: 72 years). A higher percentage of patients with gastric cancer had a history of smoking than those with breast cancer (72.4% vs 15.8%). All patients had received at least two prior chemotherapy regimens. Eighteen patients with gastric cancer had received immune checkpoint inhibitor (ICI) therapy prior to T-DXd treatment. Approximately half (49/101, 48.5%) of patients with breast cancer had a history of thoracic radiotherapy. As per the evaluation by the ILD-AC radiologists, 18 (13.8%) patients (10 with breast cancer and 8 with gastric cancer) had pre-existing interstitial pneumonia, including any interstitial shadow in lung CT scan (e.g., chronic fibrotic interstitial pneumonia) or interstitial lung abnormalities (not including gravity-dependent opacity [12]) at baseline. While no patients with gastric cancer had radiation pneumonitis or radiation pulmonary fibrosis prior to T-DXd treatment, 32 (31.7%) patients with breast cancer had these pre-existing conditions.

**Table 2:** Patient characteristics

Characteristic	Gastric cancer (*n* = 29)	Breast cancer (*n* = 101)	Total (*N* = 130)
*Age, years*			
Median (range)	72 (48–86)	62 (37–82)	64.5 (37–86)
*Age group*			
≥ 65 years	24 (82.8)	41 (40.6)	65 (50.0)
≥ 75 years	8 (27.6)	8 (7.9)	16 (12.3)
*Sex*			
Female	3 (10.3)	98 (97.0)	101 (77.7)
*Smoking history*			
Yes^a^	21 (72.4)	16 (15.8)	37 (28.5)
No	7 (24.1)	73 (72.3)	80 (61.5)
Unknown	1 (3.4)	12 (11.9)	13 (10.0)
*Number of prior chemotherapy regimens*^*b*^			
Median (range)	3 (2–7)	5.5 (2–22)	5 (2–22)
*History of prior immune check point inhibitor*			
Yes	18 (62.1)	0 (0.0)	18 (13.8)
*History of thoracic radiotherapy*			
Yes	2 (6.9)	49 (48.5)	51 (39.2)
*CT findings prior to T-DXd treatment*^*c*^			
Pre-existing IP^d^	8 (27.6)	10 (9.9)	18 (13.8)
Radiation pneumonitis or radiation pulmonary fibrosis	0 (0.0)	32 (31.7)	32 (24.6)
Lymphangitic carcinomatosis	1 (3.4)	6 (5.9)	7 (5.4)
Pleural effusion	3 (10.3)	21 (20.8)	24 (18.4)
Multiple lung metastases	6 (20.7)	45 (44.6)	51 (39.2)

Data are shown as *n* (%) unless otherwise stated

*CT* computed tomography, *ILD* interstitial lung disease, *IP* interstitial pneumonia, *T-DXd* trastuzumab deruxtecan

^a^Both past smokers and current smokers

^b^Excluding one patient who had received an unknown number of prior chemotherapy regimens

^c^Based on the evaluation by the ILD adjudication committee members (radiologists)

^d^Any interstitial shadow on the lung CT scan including chronic fibrotic interstitial pneumonia, or interstitial lung abnormalities; not including gravity-dependent opacity

### Imaging pattern and severity of ILD/pneumonitis

**Fig. 2:**
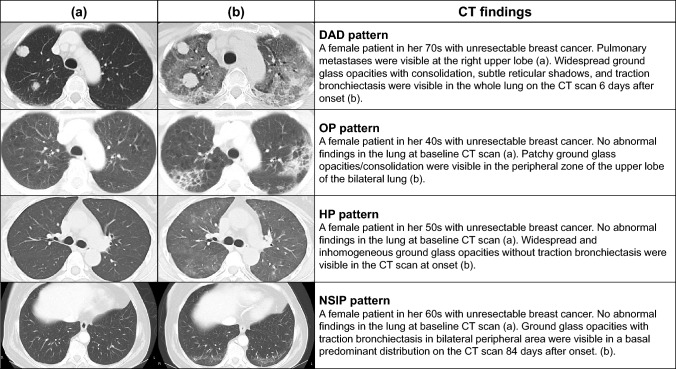
Representative images of DAD, OP, HP, and NSIP patterns of T-DXd-related ILD/pneumonitis. *CT* computed tomography, *DAD* diffused alveolar damage, *HP* hypersensitivity pneumonitis, *ILD/pneumonitis* interstitial lung disease or pneumonitis, *NSIP* non-specific interstitial pneumonia, *OP* organizing pneumonia, *T-DXd* trastuzumab deruxtecan

The imaging patterns of T-DXd-related ILD/pneumonitis cases were categorized and tabulated by the most severe grade reached (Table 3). Given the small number of T-DXd-related ILD/pneumonitis in patients with gastric cancer in this report, the imaging patterns of T-DXd-related ILD/pneumonitis cases were tabulated by using all patients with T-DXd-related ILD/pneumonitis cases. The most common pattern types observed were OP (63.1%), HP (16.9%), and DAD patterns (14.6%). NSIP patterns were observed in 3.1% of cases, and the remaining 2.3% of cases had other patterns (such as radiation recall, non-cardiogenic pulmonary edema pattern, and extremely slight shadows). The majority (100/130, 76.9%) of T-DXd-related ILD/pneumonitis cases were not severe, while 30 (23.1%) cases had a severity grade of 3 or higher. There were 11 cases (8.5%) for which the outcome was death, of which eight (72.7%) had DAD patterns and three (27.3%) had OP patterns. Representative CT images of cases with a DAD pattern, an OP pattern, an HP pattern, and an NSIP pattern are shown in Fig. 2. There were four cases where the imaging pattern changed over time: indeterminant faint ground glass opacities changed to an OP pattern (*n* = 2), an originally OP pattern changed to a DAD pattern (*n* = 1), and an HP pattern changed to an OP pattern (*n* = 1).

**Table 3:** Imaging pattern by the worst grade of ILD/pneumonitis

Imaging pattern	Grade 1	Grade 2	Grade 3	Grade 4	Grade 5	Total
Total	55 (42.3)	45 (34.6)	17 (13.1)	2 (1.5)	11 (8.5)	130 (100.0)
OP	43 (78.2)	28 (62.2)	7 (41.2)	1 (50.0)	3 (27.3)^a^	82 (63.1)
HP	9 (16.4)	12 (26.7)	1 (5.9)	0 (0.0)	0 (0.0)	22 (16.9)
DAD	0 (0.0)	1 (2.2)	9 (52.9)	1 (50.0)	8 (72.7)	19 (14.6)
NSIP	2 (3.6)	2 (4.4)	0 (0.0)	0 (0.0)	0 (0.0)	4 (3.1)
Other^b^	1 (1.8)	2 (4.4)	0 (0.0)	0 (0.0)	0 (0.0)	3 (2.3)

Data are shown as *n* (%)

*DAD* diffuse alveolar damage, *HP* hypersensitivity pneumonitis, *ILD/pneumonitis* interstitial lung disease or pneumonitis, *NSIP* non-specific interstitial pneumonia, *OP* organizing pneumonia

^a^Including ILD/pneumonitis and other diseases such as massive pleural effusion due to progression of disease or concurrent *Pneumocystis* pneumonia

^b^Including radiation recall, non-cardiogenic pulmonary edema pattern, and cases where a definitive diagnosis of ILD/pneumonitis could not be made because of very slight shadows

### ILD/pneumonitis event outcomes and response to corticosteroids

**Fig. 3:**
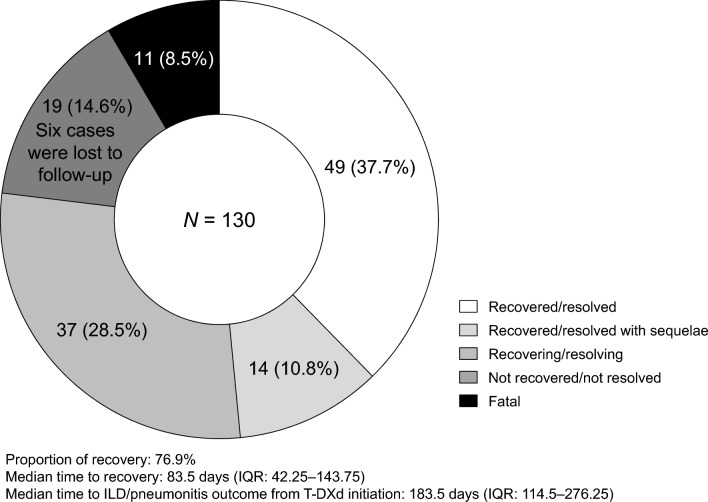
The overall proportion of ILD/pneumonitis outcomes. The proportion of recovery is the percentage of patients with the outcomes recovered/resolved, recovered/resolved with sequelae, or recovering/resolving. *ILD/pneumonitis* interstitial lung disease or pneumonitis, *IQR* interquartile range, *T-DXd* trastuzumab deruxtecan

**Fig. 4:**
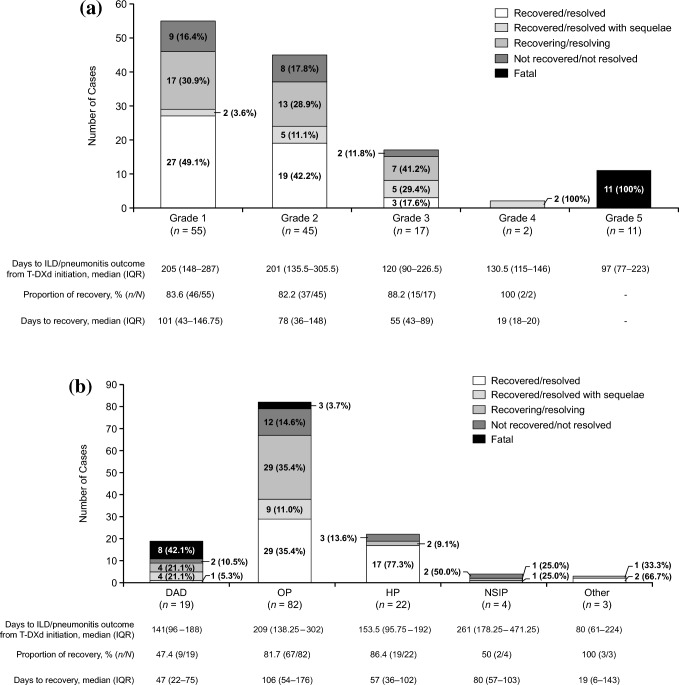
The proportion of ILD/pneumonitis outcomes by **a** worst Common Terminology Criteria for Adverse Events grade and **b** imaging pattern. The proportion of recovery is the percentage of patients with the outcomes recovered/resolved, recovered/resolved with sequelae, or recovering/resolving. In panel a, among the nine cases of grade 1 ILD/pneumonitis who did not recover, four cases were lost to follow-up. The eight cases of grade 2 ILD/pneumonitis with no recovery remained in follow-up, and the two cases of grade 3 ILD/pneumonitis who had not recovered were lost to follow-up. In panel b, the two cases with a DAD pattern and who had not recovered were lost to follow-up. Among the 12 cases with an OP pattern and who had not recovered, four were lost to follow-up. Those who had not recovered and had an HP pattern or an NSIP pattern remain in follow-up. *DAD* diffused alveolar damage, *HP* hypersensitivity pneumonitis, *ILD/pneumonitis* interstitial lung disease or pneumonitis, *IQR* interquartile range, *NSIP* non-specific interstitial pneumonia, *OP* organizing pneumonia, *T-DXd* trastuzumab deruxtecan

Table 1 shows the definition of ILD/pneumonitis event outcomes. The overall ILD/pneumonitis outcomes are shown in Fig. 3. The median time from T-DXd administration to ILD/pneumonitis outcome was 183.5 days (IQR: 114.5–276.25 days). The proportion of recovery was 76.9% at the time of data cutoff on February 27, 2022, which included the outcomes of recovered (49/130, 37.7%), recovered with sequelae (14/130, 10.8%), and recovering (37/130, 28.5%). The median time to recovery (including all recovered, recovered with sequelae, and recovering outcomes) was 83.5 days (IQR: 42.25–143.75 days). The ILD/pneumonitis outcomes stratified by the most severe CTCAE grade are shown in Fig. 4a. The proportions of recovery for grade 1, 2, 3, and 4 events were 83.6%, 82.2%, 88.2%, and 100%, respectively. Eight of the 11 fatal cases (72.7%) had DAD patterns (Online Resource 1). When stratified by imaging pattern, worse outcomes occurred in cases with DAD patterns (Fig. 4b): 8/19 (42.1%) of cases with DAD patterns had a fatal outcome. Cases with OP patterns and HP patterns had higher proportions of recovery (67/82, 81.7% and 19/22, 86.4%, respectively).

**Fig. 5:**
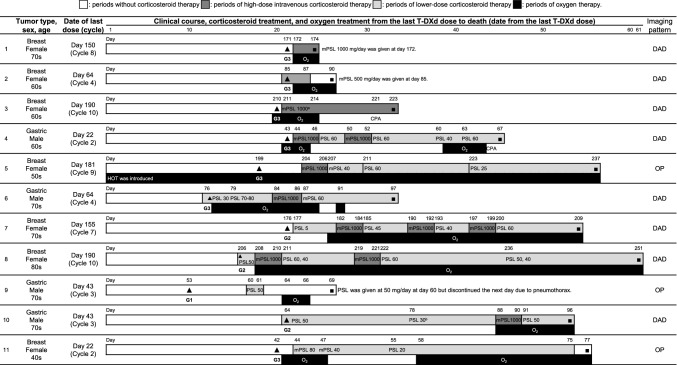
Clinical course of grade 5 ILD/pneumonitis cases. Black triangles indicate the time of onset for adjudicated ILD/pneumonitis cases. Black boxes indicate times of death. White bars indicate periods without corticosteroid therapy. Dark gray bars indicate periods of high-dose intravenous corticosteroid therapy. Light gray bars indicate periods of lower-dose corticosteroid therapy. Black bars indicate periods of oxygen therapy. The adjudicated severity grade of ILD at onset is shown below the time of onset for adjudicated cases (black triangle). The daily corticosteroid doses (milligrams) are shown after the abbreviations of corticosteroids inside of dark gray and light gray bars. ^a^The detailed periods of high-dose intravenous corticosteroid therapy of Case #3 were not provided. ^b^The initial grade 2 ILD/pneumonitis of Case #10 was improved after conventional corticosteroid therapy. However, the breathing symptoms deteriorated on Day 88, the patient died on Day 96 despite receiving high-dose corticosteroid therapy. The ILD adjudication committee determined that the cause of breathing difficulties and death was because of both worsening of ILD and *Pneumocystis* pneumonia. *CPA* cyclophosphamide, *DAD* diffused alveolar damage, *G* Common Terminology Criteria for Adverse Events grade, *HOT* home oxygen therapy, *ILD/pneumonitis* interstitial lung disease or pneumonitis, *mPSL* methyl prednisolone, *O*_*2*_ oxygen therapy, *OP* organizing pneumonia, *PSL* prednisolone, *T-DXd* trastuzumab deruxtecan

The ILD/pneumonitis cases treated with corticosteroids, stratified by severity grade, are shown in Table 4. The majority of ILD/pneumonitis cases treated with corticosteroids were considered responsive to treatment (59/71, 83.1%), including 100% of grade 2, 3, and 4 cases. The clinical course, including corticosteroid use, of the 11 fatal cases is shown in Fig. 5. Symptoms of ILD/pneumonitis deteriorated in cases 1–5, and these patients died despite immediate high-dose corticosteroid therapy after ILD/pneumonitis onset. Among the fatal cases, the clinical course for some cases (*n* = 3) indicated that high-dose corticosteroids were not initiated immediately, despite grade 3 severity ILD/pneumonitis at the time of onset.

**Table 4:** Corticosteroid responsiveness by the worst grade of ILD/pneumonitis

Outcomes	Grade 1	Grade 2	Grade 3	Grade 4	Grade 5	Total
*Total*	55	45	17	2	11	130
*ILD/pneumonitis cases receiving corticosteroids*	12	34	17	2	11	76
*Corticosteroid responsiveness*^*a*^						
Evaluated	9	33	16	2	11	71
Yes	8 (88.9)	33 (100.0)	16 (100.0)	2 (100.0)	0 (0.0)	59 (83.1)
No	1^b^ (11.1)	0 (0.0)	0 (0.0)	0 (0.0)	11 (100.0)	12 (16.9)
*Corticosteroid responsiveness not evaluable*^*c*^	3	1	1	0	0	5

Data are shown as *n* (%)

*ILD/pneumonitis* interstitial lung disease or pneumonitis

^a^Corticosteroid responsiveness was judged by pulmonologists from the ILD adjudication committee based on the patient’s clinical course, radiological images, and other relevant data for adjudication

^b^Although corticosteroid treatment was initiated 6 days after the onset of ILD/pneumonitis, the patient died 38 days after the onset of ILD/pneumonitis because of disease progression

^c^The reasons for not evaluating the corticosteroid responsiveness included a lack of images/clinical data for follow-up after onset of ILD/pneumonitis, the patient died during tapering, the patient received corticosteroids for other reasons (e.g., brain edema)

## Discussion

T-DXd is becoming more broadly used for HER2-expressing or mutant cancers worldwide. As more patients use T-DXd in the future, the management of potential ILD/pneumonitis in T-DXd-treated patients is of prime importance. During the post-marketing period between May 25, 2020 and February 24, 2022, approximately 3000 patients received T-DXd; 287 of these patients (breast cancer: *n* = 207, gastric cancer: *n* = 80) were reported by their physician as potential ILD/pneumonitis cases, and 130 cases were evaluated as T-DXd-related ILD/pneumonitis (breast cancer: *n* = 101, gastric cancer: *n* = 29). The majority of cases adjudicated as T-DXd-related ILD/pneumonitis were grade 1 or 2, and 23.1% of cases were grade 3 or higher. The clinical outcomes varied by imaging pattern, suggesting that cases with DAD patterns have a poor prognosis and those with OP and HP patterns have favorable outcomes [11]. In addition, most ILD/pneumonitis cases that were treated with corticosteroids were considered responsive to treatment.

Although the pooled analysis of nine clinical trials reported the frequency of T-DXd-related ILD/pneumonitis to be 15.4% in patients treated with T-DXd [10], the currently available surveillance data suggest that the overall incidence of adjudicated T-DXd-related ILD/pneumonitis may be lower than the incidence previously reported. However, many patients enrolled in this surveillance were still within the observation period, meaning that not all potential ILD/pneumonitis cases have yet been identified or adjudicated; moreover, the outcomes of some ILD/pneumonitis cases were still being followed up at the data cutoff (February 27, 2022). Further investigation is needed to evaluate the overall incidence of adjudicated T-DXd-related ILD/pneumonitis in the post-marketing setting. Moreover, the lower rates of ILD/pneumonitis in patients with gastric cancer may also reflect the 6-month gap between approval of T-DXd for breast cancer and for gastric cancer in Japan. Importantly, we observed no noticeable increase in the incidence of ILD/pneumonitis compared with the incidence reported by the previous pooled analysis [10].

Many of the patients with gastric cancer and T-DXd-related ILD/pneumonitis had received prior treatment with ICIs (18/29, 62.1%). These patients did not have an earlier onset of ILD/pneumonitis or worse outcomes compared with those who had not received ICIs. The previous pooled analysis found no connection between prior treatment with ICIs and the frequency of ILD/pneumonitis [10]. Furthermore, this report was not designed to evaluate risk factors for ILD/pneumonitis. Therefore, it is currently unknown what effect this factor may have on ILD/pneumonitis risk.

Among the 130 adjudicated T-DXd-related ILD/pneumonitis cases that were evaluated, most cases (76.9%) had a low severity grade (1 or 2), which is similar to the previous pooled analysis [10]. We observed a spectrum of patterns including OP, HP, and DAD patterns. A similarly wide range of patterns was reported in earlier clinical trials [13] and studies of other anti-cancer drugs [11, 14]. No imaging pattern specific to T-DXd-related ILD/pneumonitis was observed in this report. We observed that the majority (8/11, 72.7%) of fatal ILD/pneumonitis cases had DAD patterns; this is consistent with previous reports of other anti-cancer drugs [11, 15–19]. ILD/pneumonitis cases with DAD patterns often respond poorly to corticosteroid treatment [11], and some of the patients with DAD patterns in this report died even after receiving high-dose corticosteroid treatment immediately after diagnosis. However, in the absence of other effective treatment options, high-dose corticosteroid therapy should be initiated as soon as possible. Careful monitoring, prompt diagnosis, and immediate treatment are important for ILD management [20] and may potentially help prevent poor outcomes of patients with DAD patterns.

In the early phase of clinical trials of T-DXd, ILD/pneumonitis was identified as an important risk; the guidance for managing T-DXd-related ILD/pneumonitis was created with reference to established guidance for ILD/pneumonitis induced by other anti-cancer drugs and was used during the T-DXd clinical trials [1, 3–9, 21–24]. However, many of the non-serious ILD/pneumonitis cases did not have a reported outcome by the end of the study and the response of these cases to corticosteroid therapy was not evaluated [13]. Thus, the reversibility of T-DXd-related ILD/pneumonitis and its responsiveness to corticosteroid therapy is not clearly understood. A statement has since been added to the protocol, clarifying that all ILD/pneumonitis events, regardless of severity or seriousness, must be followed until resolution, including after drug discontinuation. Furthermore, evaluation of the AE outcomes in the T-DXd clinical trials was at the investigator’s discretion. Therefore, in the post-marketing setting, the ILD-AC and the study sponsor established definitions of T-DXd-related ILD/pneumonitis outcomes so that the committee could evaluate outcomes consistently. Cases were followed until the case was considered either recovered or recovered with sequelae, or for up to 12 months after the onset of ILD/pneumonitis. For the cases whose outcome was recovering (*n* = 37) or not recovered (*n* = 19), the final outcome will be followed up, and a better outcome can be expected for some cases.

The approved labels for T-DXd include warnings regarding ILD/pneumonitis and guidance for its management, suggesting the use of corticosteroids [25–27]. To our knowledge, there has been no published report regarding the effectiveness of corticosteroids for treating T-DXd-related ILD/pneumonitis. A non-clinical report suggests T-DXd-related ILD/pneumonitis is caused by lung inflammation [28]. Therefore, treatment of ILD/pneumonitis involves suppression of inflammation and prevention of irreversible fibrosis [29]. The timing of corticosteroids influences their effectiveness; treatment is considered to be most effective when administered soon after onset, during the inflammatory phase of the condition [30]. High-dose corticosteroid treatment has a strong anti-inflammatory effect that is induced by non-genomic mechanisms [31], and is recommended for severe drug-induced ILD/pneumonitis [11, 20]. In this report, most (83.1%) of the cases evaluated for response to corticosteroids were considered responsive. However, in 5 of 11 fatal cases, patients rapidly deteriorated and died despite immediate administration of high-dose corticosteroids. Rapid deterioration can be difficult to identify in cases with faint shadows on imaging scans, but immediate treatment with high-dose corticosteroids is recommended if bilateral diffuse opacities, dyspnea, or a significant decrease in oxygen saturation from baseline occurs [20]. Other studies of fatal ILD/pneumonitis cases have shown the importance of following the current ILD/pneumonitis guidance [20, 32–34]. Our findings show that T-DXd-induced ILD/pneumonitis can progress rapidly and reiterate the importance of careful monitoring of patients receiving T-DXd to provide prompt treatment of severe (grades 3 and above) ILD/pneumonitis cases.

In Japan, rechallenge with T-DXd was not permitted for patients who experienced any grades of ILD/pneumonitis during the period of this surveillance; therefore, the safety of rechallenge with T-DXd treatment was not evaluated in this report. However, as of November 2022, T-DXd rechallenge after complete resolution of grade 1 ILD/pneumonitis was allowed. The safety of resuming treatment with T-DXd in these patients will need to be assessed in the future.

We acknowledge the limitations of this report: this was an ad hoc exploratory analysis. Corticosteroid use (such as duration and route of administration) may be inconsistently recorded on clinical report forms. Future studies on the association between corticosteroid therapy and T-DXd-related ILD/pneumonitis outcomes are needed. Additionally, the evaluated data and cases with an earlier onset of ILD/pneumonitis were considered to have a bias toward serious ILD/pneumonitis cases for two reasons: 1) serious ILD/pneumonitis cases were prioritized for sending to the ILD-AC, and 2) cases that had an earlier onset of ILD/pneumonitis were ready for adjudication before those with late onset. Therefore, our findings may not be representative of all T-DXd-related ILD/pneumonitis cases in Japan and should be carefully interpreted. The incidence of T-DXd-related ILD/pneumonitis cases by severity grade and the time to ILD/pneumonitis onset will be summarized and reported when the adjudication of all potential ILD/pneumonitis cases is completed.

In conclusion, this report is the first to evaluate the adjudicated T-DXd-related ILD/pneumonitis cases in a Japanese post-marketing environment. The relationship between imaging pattern type and ILD/pneumonitis severity was consistent with previous reports, suggesting that cases with DAD patterns have poor outcomes. Evaluating a larger real-world dataset may further identify predictors of clinical outcome.

### Supplementary Information

Below is the link to the electronic supplementary material.

Supplementary file1 (DOCX 298 KB)

## Data Availability

The data supporting the findings in this study are available within the paper, in the corresponding Online Resource, or upon reasonable request from the corresponding author.
