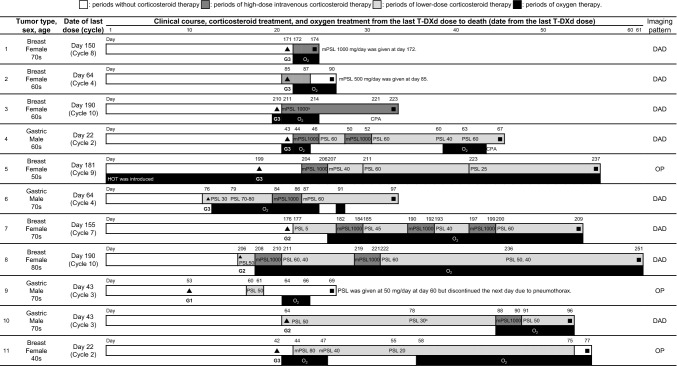# Correction to: Clinical and imaging features of interstitial lung disease in cancer patients treated with trastuzumab deruxtecan

**DOI:** 10.1007/s10147-023-02427-6

**Published:** 2023-10-28

**Authors:** Tomohisa Baba, Masahiko Kusumoto, Terufumi Kato, Yasuyuki Kurihara, Shinichi Sasaki, Katsunori Oikado, Yoshinobu Saito, Masahiro Endo, Yutaka Fujiwara, Hirotsugu Kenmotsu, Masafumi Sata, Toshimi Takano, Ken Kato, Koji Hirata, Tomomi Katagiri, Hanako Saito, Kazuyoshi Kuwano

**Affiliations:** 1 Department of Respiratory Medicine, Kanagawa Cardiovascular and Respiratory Center, 6-16-1 Tomiokahigashi, Kanazawa-ku, Yokohama-shi, Kanagawa 236-0051 Japan; 2 Department of Diagnostic Radiology, National Cancer Center Hospital, 5-1-1 Tsukiji, Chuo-ku, Tokyo, 104-0045 Japan; 3 Department of Thoracic Oncology, Kanagawa Cancer Center, 2-3-2 Nakao, Asahi-ku, Yokohama-shi, Kanagawa 241-8515 Japan; 4 Department of Radiology, St. Luke’s International Hospital, 9-1 Akashi-cho, Chuo-ku, Tokyo, 104-8560 Japan; 5 Department of Respiratory Medicine, Juntendo University Urayasu Hospital, 2-1-1 Tomioka, Urayasu-shi, Chiba 279-0021 Japan; 6 Department of Diagnostic Imaging, The Cancer Institute Hospital of Japanese Foundation for Cancer Research, 3-8-31 Ariake, Koto, Tokyo, 135-8550 Japan; 7 Department of Pulmonary Medicine and Oncology, Nippon Medical School, 1-1-5 Sendagi, Bunkyo-ku, Tokyo, 113-8602 Japan; 8 Division of Diagnostic Radiology, Shizuoka Cancer Center, 1007 Shimonagakubo, Nagaizumi-cho, Sunto-gun, Shizuoka 411-8777 Japan; 9 Department of Thoracic Oncology, Aichi Cancer Center Hospital, 1-1 Shikoden, Chikusa-ku, Nagoya-shi, Aichi 464-8681 Japan; 10 Division of Thoracic Oncology, Shizuoka Cancer Center, 1007 Shimonagakubo, Nagaizumi-cho, Sunto-gun, Shizuoka, 411-8777 Japan; 11 Division of Respiratory Medicine, Department of Internal Medicine, Jichi Medical University, 3311-1 Yakushiji, Shimotsuke-shi, Tochigi 329-0498 Japan; 12 Breast Medical Oncology Department, The Cancer Institute Hospital of Japanese Foundation for Cancer Research, 3-8-31 Ariake, Koto, Tokyo, 135-8550 Japan; 13 Department of Head and Neck, Esophageal Medical Oncology, National Cancer Center Hospital, 5-1-1 Tsukiji, Chuo-ku, Tokyo, 104-0045 Japan; 14 Clinical Safety and Pharmacovigilance Division, Medical Safety Department, Daiichi Sankyo Co., Ltd., 3-5-1, Nihonbashi Honcho, Chuo-ku, Tokyo, 103-8426 Japan; 15 Division of Respiratory Diseases, Department of Internal Medicine, The Jikei University School of Medicine, 3-25-8 Nishi-Shinbashi, Minato-ku, Tokyo, 105-8461 Japan

**Correction to: International Journal of Clinical Oncology** 10.1007/s10147-023-02414-x

In this article, the wrong figure appeared as Fig. 5; the Fig. 5 should have appeared as shown below:

**Figure:**