# Supplementary material for: Clinical and imaging features of interstitial lung disease in cancer patients treated with trastuzumab deruxtecan
Source: Int J Clin Oncol. 2023 Oct 3;28(12):1585–96. doi: 10.1007/s10147-023-02414-x (PMC10687185; doi:10.1007/s10147-023-02414-x)
Supplement: Supplementary file 1 — Supplementary file1 (DOCX 298 KB) [file 10147_2023_2414_MOESM1_ESM.docx]

*International Journal of Clinical Oncology*

**Online Resource**

**Clinical and imaging features of interstitial lung disease in cancer patients treated with trastuzumab deruxtecan**

Tomohisa Baba, Masahiko Kusumoto, Terufumi Kato, Yasuyuki Kurihara, Shinichi Sasaki, Katsunori Oikado, Yoshinobu Saito, Masahiro Endo, Yutaka Fujiwara, Hirotsugu Kenmotsu, Masafumi Sata, Toshimi Takano, Ken Kato, Koji Hirata, Tomomi Katagiri, Hanako Saito, Kazuyoshi Kuwano

## Corresponding author:

Tomohisa Baba

Department of Respiratory Medicine, Kanagawa Cardiovascular and Respiratory Center, 6-16-1 Tomiokahigashi, Kanazawa-ku, Yokohama, Kanagawa 236-0051, Japan

Email: baba.19049@kanagawa-pho.jp


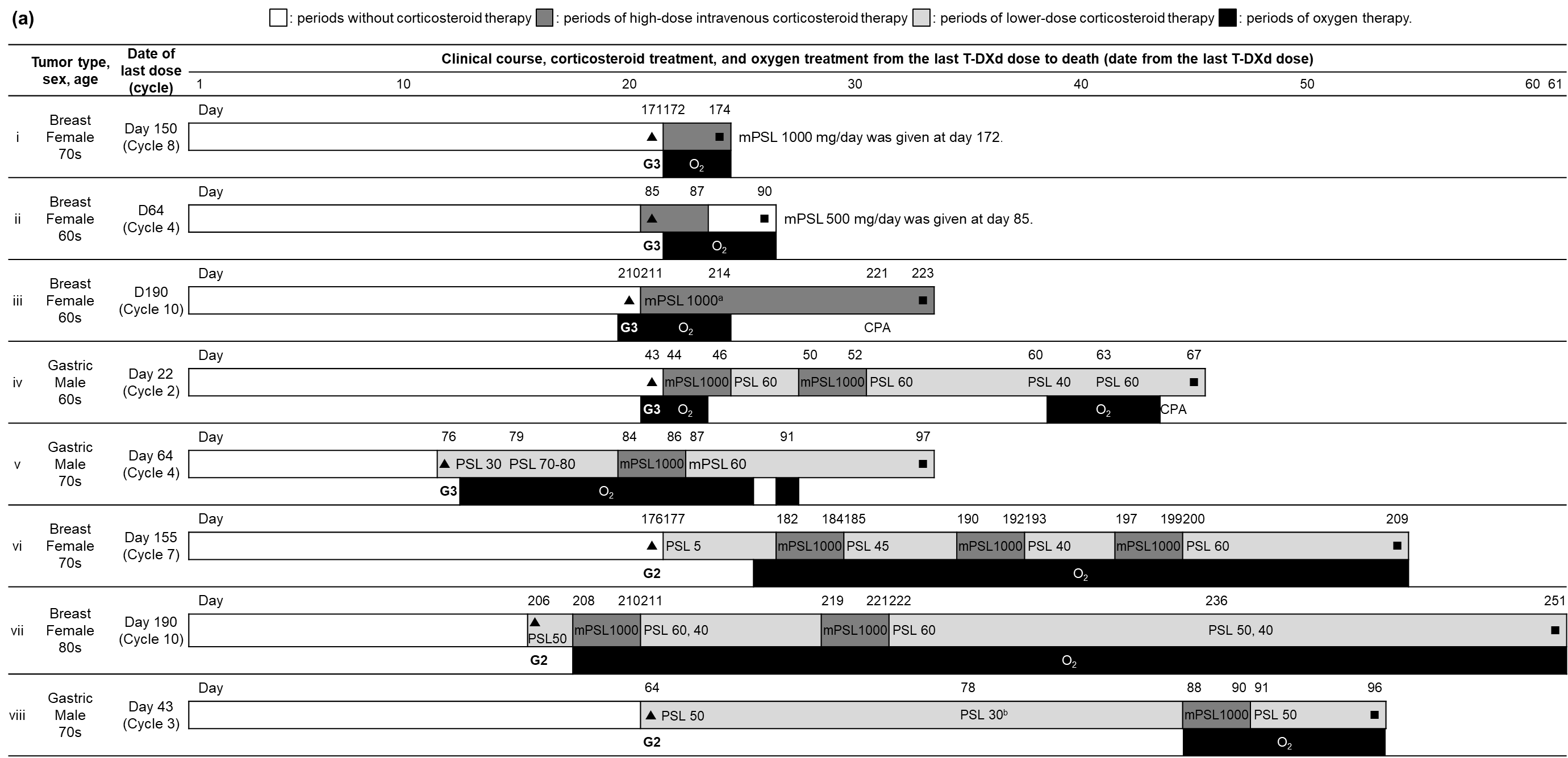


**
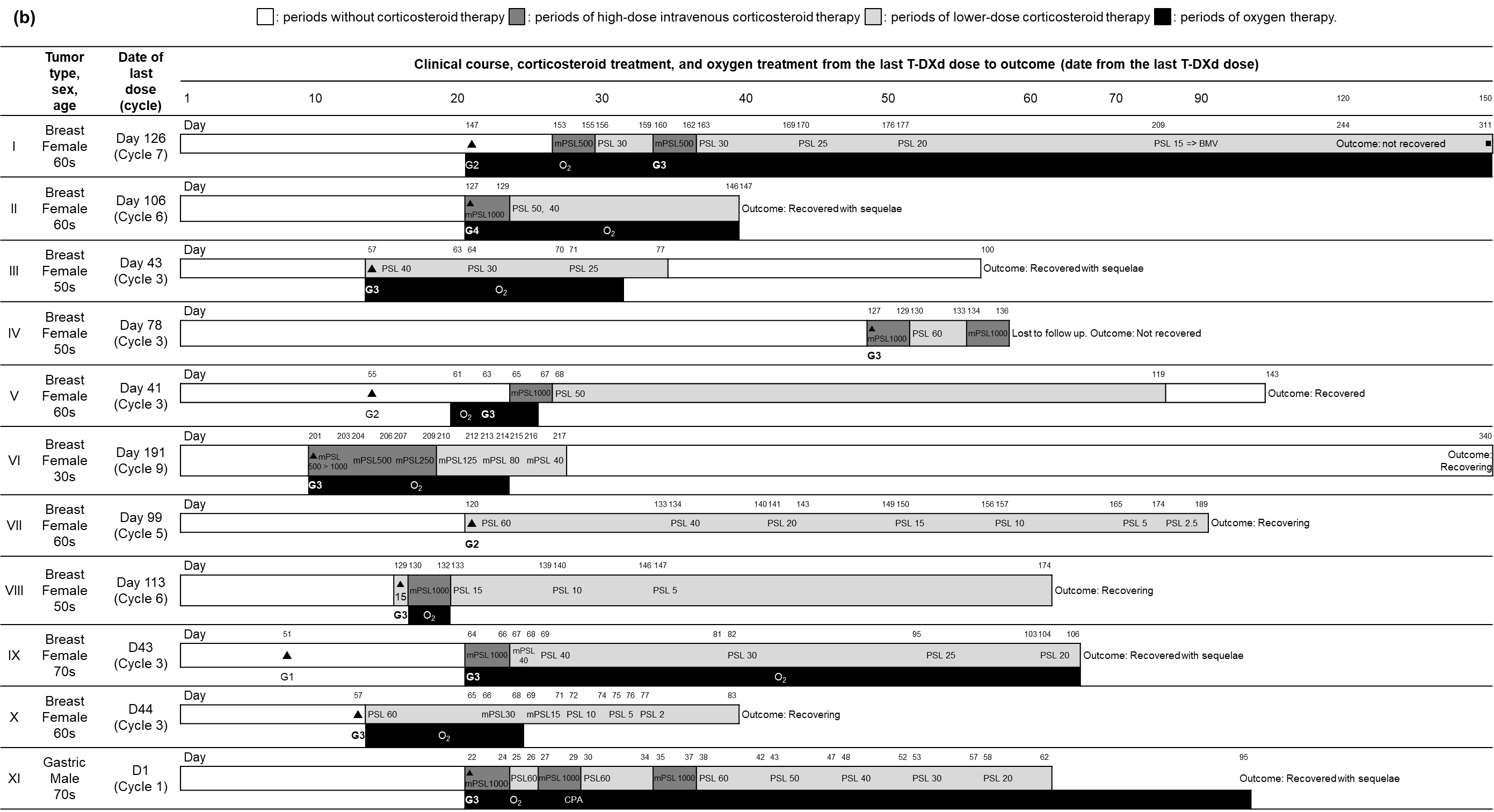
**

**Online Resource 1** Clinical course of ILD/pneumonitis cases with DAD patterns

(a) Grade 5 cases with DAD patterns (*n* = 8).

Case number in Online resource (a) corresponds to the case number in Fig. 5 as follows: #i = #1, #ii = #2, #iii = #3, #iv = #4, #v = #6, #vi = #7, #vii = #8, and #viii = #10, respectively.

(b) Grade 2–4 cases with DAD patterns (*n* = 11).

Case number in Online resource (b) has no correspondence to the case number in Fig. 5.

Black triangles (▲) indicate the time of onset for adjudicated ILD/pneumonitis cases. Black boxes (■) indicate times of death. White bars indicate periods without corticosteroid therapy. Dark gray bars indicate periods of high-dose intravenous corticosteroid therapy. Light gray bars indicate periods of lower-dose corticosteroid therapy. Black bars indicate periods of oxygen therapy. The adjudicated severity grade of ILD/pneumonitis at onset is shown below the time of onset for adjudicated cases (▲). The daily corticosteroid doses (milligrams) are shown after the abbreviations of corticosteroids inside the green and light blue bars.

^a^The detailed periods of high-dose intravenous corticosteroid therapy of Case #iii were not provided.

^b^The initial Grade 2 ILD/pneumonitis of Case #viii was improved after conventional corticosteroid therapy. However, the breathing symptoms deteriorated on Day 88, the patient died on Day 96 despite receiving high-dose corticosteroid therapy. The ILD adjudication committee determined that the cause of breathing difficulties and death was because of both worsening of ILD and *Pneumocystis* pneumonia.

*CPA* cyclophosphamide, *BMV* betamethasone valerate, *DAD* diffused alveolar damage, *G* Common Terminology Criteria for Adverse Events grade, *ILD/pneumonitis* interstitial lung disease/pneumonitis, *mPSL* methyl prednisolone, *O_2_* home oxygen therapy, *PSL* prednisolone, *T-DXd* trastuzumab deruxtecan
